# Scientific Investigation

**Published:** 1875-02

**Authors:** 


SCIENTIFIC INVESTIGATION.—It so happens that in order to know certain things which cannot be known save by operating on a living creature, it has been deemed necessary to make careful observations of some of the lower animals. In some instances pain is inflicted, of course. But surgeons, since the days of Galen, have considered that the end justified the means, and that if man could be saved it were no sin to bring suffering to inferior creatures. Of course, the humane surgeon will not inflict pain needlessly; it is, however, folly to say that useful investigations shall not go on because of the trifling suffering which some dog or cat or rabbit may endure. Of the knowledge gained for mankind by means of experiments on living animals, the most remarkable is: The circulation of the blood; the chemical phenomena of respiration; the power of artificial respiration to maintain or restore life under seemingly desperate circumstances; transfusion of blood whereby many patients have been rescued from the brink of the grave; all that is known of the digestive processes in health and disease; the entire range of our information (even yet incomplete) concerning the functions and disorders of the nervous system; the successful surgical treatment of aneurism; the discovery of the regeneration of bone from its periosteal covering, leading to the preservation of thousands of limbs which would formerly have been amputated; the ascertainment of the mode of action of snake-poison; the demonstration of the origin, and consequently of the means of preventing various parasitic diseases; and the list might be much farther prolonged, including nearly all that we have positively learned and all that we hope to learn about the physiological actions of the drugs on which we rely in the treatment of disease. While we are as earnest in our condemnation of all wanton cruelty to animals, and rejoice to see that humanity is fast taking the place of thoughtlessness and barbarity in regard to the treatment which they should receive at the hands of man, we cannot agree with those who would thus put an end to scientific investigation of a very important and useful kind. The accident which tore open the stomach of Alexis St. Martin was the most useful one which ever befell a man, as it enabled his physician to watch the processes of digestion—a thing never before accomplished. As the accidents which befall humanity are not sufficient for all the purposes of investigation, we must avail ourselves of such information as can be gathered from domestic animals—the beasts of the field and the fowls of the air; and we can see no reason why we may not fit ourselves to prolong human life by practicing upon animals, which would not apply with equal force to the slaughtering of cattle for food. Shall we say that the transfusion of the warm blood of a lamb into the veins of a dying man is cruel, because the puncture induces trifling pain? This is sentimentality; it certainly is not good sense.

---

HEADACHE.—Dr. Brunton tells us that the administration of a brisk purgative, or small doses of Epsom salts, thrice a day, is a most effectual remedy for frontal headache when combined with constipation; but if the bowels be regular, the morbid processes on which it depends seem to be checked and the headache removed even more effectually by one or two drops of nitro-hydrochloric acid in a wine glass of water, or by a little ammonia in water, given before meals. If the headache be immediately above the eyebrows, the acid is best; but if it be a little higher up, just where the hair begins, the alkalies appear to be the more serviceable. At the same time that the headache is removed, the feelings of sleepiness and weariness, which frequently lead the patients to complain that they rise up more tired than they lie down, generally disappear.

